# One Digital Health: A Unified Framework for Future Health Ecosystems

**DOI:** 10.2196/22189

**Published:** 2021-02-05

**Authors:** Arriel Benis, Oscar Tamburis, Catherine Chronaki, Anne Moen

**Affiliations:** 1 Faculty of Technology Management Holon Institute of Technology Holon Israel; 2 Faculty of Digital Medical Technologies Holon Institute of Technology Holon Israel; 3 Department of Veterinary Medicine and Animal Productions University of Naples Federico II Naples Italy; 4 HL7 Europe Brussels Belgium; 5 Faculty of Medicine Institute for Health and Society University of Oslo Oslo Norway

**Keywords:** One Health, digital health, eHealth, medicine, veterinary medicine, environmental monitoring, education, patient engagement, citizen science, health care industry, population health management, data science, COVID-19

## Abstract

One Digital Health is a proposed unified structure. The conceptual framework of the One Digital Health Steering Wheel is built around two keys (ie, One Health and digital health), three perspectives (ie, individual health and well-being, population and society, and ecosystem), and five dimensions (ie, citizens’ engagement, education, environment, human and veterinary health care, and Healthcare Industry 4.0). One Digital Health aims to digitally transform future health ecosystems, by implementing a systemic health and life sciences approach that takes into account broad digital technology perspectives on human health, animal health, and the management of the surrounding environment. This approach allows for the examination of how future generations of health informaticians can address the intrinsic complexity of novel health and care scenarios in digitally transformed health ecosystems. In the emerging hybrid landscape, citizens and their health data have been called to play a central role in the management of individual-level and population-level perspective data. The main challenges of One Digital Health include facilitating and improving interactions between One Health and digital health communities, to allow for efficient interactions and the delivery of near–real-time, data-driven contributions in systems medicine and systems ecology. However, digital health literacy; the capacity to understand and engage in health prevention activities; self-management; and collaboration in the prevention, control, and alleviation of potential problems are necessary in systemic, ecosystem-driven public health and data science research. Therefore, people in a healthy One Digital Health ecosystem must use an active and forceful approach to prevent and manage health crises and disasters, such as the COVID-19 pandemic.

## Introduction and Background

All science fields are strongly interconnected to each other, and each health specialty depends on others. Yet academics, researchers, and practitioners are often confined to their own knowledge, language, and expertise silos. This issue is paradoxically enhanced by the existing abundance of knowledge; large amounts of big data and the cloud age have supported short-term research in numerous domains [1-3].

One Health is an umbrella concept that encompasses all disciplines that broadly deal with human health, animal health, and the surrounding environment. The modern origins of One Health date back to 2004. One Health was introduced as part of the 12 Manhattan Principles, which called for an international, interdisciplinary approach for preventing diseases [4], specifically animal-human transmissible and communicable diseases. Therefore, systematic perspectives on life sciences and the environment (ie, interactions and coinfluences within the environment) were brought together to design and implement programs, policies, and regulations for achieving better public health outcomes. The World Health Organization (WHO) has associated One Health with sustainable development goals [5,6]. One Health involves the evaluation and monitoring of the impact of environmental hazards on health care systems, public health, biodiversity, and food security. In summary, One Health encompasses the interconnections between humans, animals, plants, and the global ecological environment [7-10]. Therefore, One Health research requires close collaboration among health practitioners, public health workers, biologists, and environmental science specialists. One of the main aims of One Health research is to understand how potential vector-borne diseases (eg, a highly pathogenic avian influenza) spread and how they can be controlled [11,12].

The evolution of digital technology has resulted in evidence-based, accessible, upper-level, and holistic methods that are capable of accelerating biomedical research and enhancing public health efficacy. Moreover, this transformation has rapidly enhanced scientific knowledge development and improved health education, personalized clinical care, and citizen science. This has led to the growth and advancement of scientific knowledge, as well as overlaps among formal, natural, and social sciences; engineering; health; medicine; and well-being. There have been calls for integrating digital technologies into the living- and society-related aspects of various fields to enhance population health, efficiency, precision, and personalization in health care delivery. Thanks to active citizens’ engagement and participation, considerable volumes of data have been generated by a multitude of systems and processed with semi–real-time data mining techniques (ie, techniques that health care data specialists have improved over time). Reflecting upon multiple perspectives during the ongoing COVID-19 pandemic can provide opportunities for revisiting and understanding the limitations of public health, human and veterinary health care, and environmental management systems [13,14].

To build upon these advances in digital technology, we introduce One Digital Health (ODH), which is a novel framework that integrates perspectives from health, care, and well-being research; health informatics research; and broad computer and data science research. ODH is based on (1) health informatics and the broadening of digital health [15]; (2) One Health [4], which provides a holistic and systemic view of health and life sciences that are prominent in human health, animal health, and ecosystem research; and (3) environmental research.

In the following sections of this viewpoint paper, we will elaborate on the ODH conceptual framework, highlight the core elements of ODH, and address specific challenges for the future. Examples of COVID-19–related aspects and how these aspects relate to the ODH framework will also be considered and discussed. We will also discuss recommendations for raising awareness, capacity building in ODH, and the proposed multidisciplinary European Federation of Medical Informatics (EFMI) ODH working group, to promote synergy across One Health scientific fields.

## What is ODH?

ODH aims to facilitate and improve collaboration among practitioners in One Health and digital health communities. This collaboration will allow both communities to benefit from efficient interactions over time and the delivery of near–real-time, data-driven contributions to systems medicine [16] and systems ecology [17]. This will also allow citizens to engage with their individual health and well-being.

ODH is comprised of three intertwined levels (ie, two enabling keys, three perspectives, and five dimensions), wherein digital technology acts a catalyst. These are described in the following sections.

**Figure 1:**
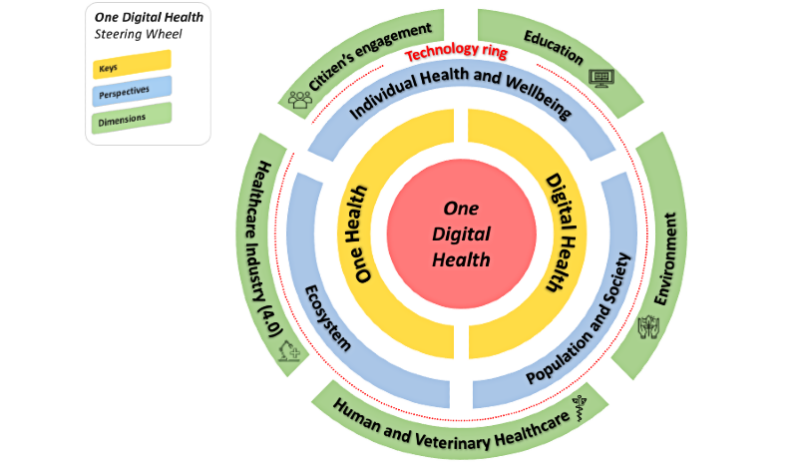
The One Digital Health Steering Wheel conceptual framework.

Figure 1 shows our conceptualization of the ODH framework, which we named the ODH Steering Wheel.

## The Two Enabling Keys: One Health and Digital Health

It is widely acknowledged that the concepts of health data, information, and knowledge refer to a person’s health and medical history [18]. Health informatics refers to the management (ie, collection, aggregation, analysis, and interpretation) of health data, information, and knowledge (ie, those related to patient care) that are made available through the deployment of digital tools [19]. Many scholars have contributed to the expansion of these original core concepts. Such contributions include (1) increasing technology adoption and performance; (2) enhancing technology safety, quality, effectiveness; and (3) improving the efficiency of care. One Health and digital health researchers seek to make their fields more comprehensive and inclusive, by studying research fields that are constantly being improved due to the development of health informatics [20]. As a result, the field of biomedical and health informatics (BMHI) “pursues the effective uses of biomedical data, information, and knowledge for scientific inquiry, problem solving, and decision making, motivated by efforts to improve human health”; and “investigates and supports reasoning, modeling, simulation, experimentation, and translation across the spectrum from molecules to populations, dealing with a variety of biological systems” [21]. In this sense, BMHI reflects manifold purposes and benefits all professionals in the health care field [22].

During the early 2000s, the internet was integrated into the day-to-day life of the general public. At the same time, eHealth emerged as a novel concept. eHealth has expanded the focus of medical informatics to include clinical information systems [23]. This has allowed the field to actively acknowledge health service customers, which are the central focus of the system. Thus, eHealth has majorly emphasized the importance of consumer health informatics in the current century [24].

eHealth-related concepts have been defined [24] and disseminated over more than 20 years [25]. Nonetheless, the massive process of digitalizing health services (ie, the process we are currently witnessing) has brought renewed focus to economics. This focus has manifested as the use of the term “digital health” [26]. This means that even though the terms “electronic something” and “digital something” are considered synonyms, the former seemingly refers to “electromagnetic stuff” from the “old times” (ie,1990s to the beginning of 2000s), whereas the latter sounds more like “advanced technologies” that fully support the daily practices of health and care. Thus, the term “digital health” is catchier than “electronic health,” which can refer to communities that have not fully integrated technology [26]. In other words, no considerable differences seem to exist between eHealth and digital health, as no paradigm shifts exist between them. Yet, digital health can be seen as a refined version of eHealth, since digital health explicitly accounts for the deployment of health data, information, knowledge, and decision support systems via digital technologies, in a context that features life and environmental aspects [23]. Digital health therefore entails (1) ubiquitously collecting and storing data, information, and knowledge to efficiently deliver health care; (2) making health and medicine more personalized and precise; (3) pursuing goals that are related to health promotion, well-being, and efficient self-management; (4) considering the economic dimensions of the services tendered.

The deployment of digital health interventions requires expertise from health practitioners; health researchers; and scientists in the fields of engineering, social sciences, public health, health economics, and data management [21]. It is for these reasons that we use the term “digital health” instead of “eHealth” in this viewpoint paper.

Concepts such as health, well-being, individual, population, living quality, and biological systems stand at the core of One Health practitioners’ collaborative, multisectoral, and transdisciplinary approach. One Health practitioners also aim to achieve optimal health and well-being outcomes at the local, regional, national, and global levels, by recognizing the interconnections between people, animals, plants, and their ecosystems [27-29]. People’s rising interest in the manifold competence areas of One Health have allowed them to witness the development of innovative strands of research, such as digital epidemiology and public health infodemiology research [30,31]. These new disciplines aim to improve people’s understanding of health risks, effective management, and policy decisions [32,33]. In this spirit, One Health informatics has been proposed as an approach for bringing these research topics together during public health and life sciences research. The key themes of One Health informatics are deploying big data analytics for supporting and improving public health and medical research, and addressing issues that are related to biodiversity control, disaster management, and disease monitoring (eg, the surveillance of zoonoses for determining early warning signs, prevention methods, and control methods).

One Health practitioners worldwide have been calling for engagement at a much broader, ecosystem-level scale. Systemic and cultural transformations are necessary, as they allow us to appreciate how disruptive technologies play a big role in the implementation of new practice models, which encourages new ways of thinking about health, care, and wellness. Thus, the profound impact of disruptive technologies is expected to shape the future of health systems. This is why the very idea of “health systems” should be reconsidered [34,35]. We propose that the One Health informatics frame, which includes the concepts of living, society, and digital health intervention–related expertise, should be included in One Health’s widened framework, which looks at the inextricable interconnectedness between humans, animals, and the environment [9,10]. The following aspects should be included: (1) the delivery of health care in a syndemic scenario [36]; (2) the digital transformation of human and animal health data (ie, including the concept of animal welfare) [37]; and (3) digital nature conservation (ie, biodiversity and wildlife conservation, food security, antimicrobial resistance, climate change, etc) by means of digital technology–based interventions [38].

A wide range of scientific disciplines has been called upon to work together to address emerging issues that are related to the above aspects. This holistic set of aspects and expertise requirements can be brought together under the proposed ODH framework.

## The Three Perspectives: The “Individual-Population-Ecosystem” Triad

The perspectives layer of the ODH looks at how the concept of “individual” is identified within that of “population” (ie, without distinguishing between human and animal), how a population impacts and interacts with an ecosystem, and how the ecosystem responds accordingly.

### Individual Health Care and Well-being

The emerging awareness of shared risks that face animal and human populations (ie, a concept that was originally part of the One Health approach) fosters opportunities for leveraging large amounts of unexplored data sources on zoonoses to generate new information and knowledge. The scope of the ODH includes supporting rapid and more accurate methods for detecting disease trends, outbreaks, pathogens, and causes of emergence [8,39-41]. The recognized, paramount importance of collecting digital health data to strengthen our understanding highlights the need for frameworks and tools that support and acknowledge the shift from big data to smart data. Smart data involves innovative processes that are necessary for answering pivotal questions from the health sector [33]. Addressing these issues requires new, ecosystem-driven paradigms and transformative technologies that reinforce traditional surveillance systems’ ability to prevent and control diseases. These innovations will further impact intersectoral coordination; link human and animal health data; allow for the flow of reliable information and knowledge; and promote the proper use of infrastructures, systems, and human resources for detecting outbreaks, thereby ensuring good health and well-being.

### Population and Society

Discussing populations and their societal organization does not simply mean providing a generalization of concepts that are related to personalized health care and well-being. Although we are discussing individual-level, predictive, personalized, preventive, and participatory health care [42-44], shifting to the topic of population-level, unified perspectives requires us to account for the differences between individuals. Contrary to a one-size-fits-all approach, personalized health has to take into account an individual’s variability in terms of genes, environments, digital health literacy, preferences, and lifestyles. This requires a systematic integration of knowledge (ie, knowledge about health care, veterinary care, agriculture, meteorology, climate change, environmental protection, and intelligence) [45] into novel decision-making processes. These processes include decisions about the prevention and management of all hazards and threats to public health, such as exposure to animal diseases with zoonotic potential, environmental exposures, drug adverse events, the rise of misinformation [46], instances of diseases, and disease outbreaks [47].

There is a lack of solid and recognized leadership. Therefore, establishing an ethical framework that is socially and culturally accepted will lead to thriving digital platforms that can develop and grow independently. Such platforms will evolve to deliver the needs of a business.

### Innovative Ecosystem

Due to peoples’ current understanding of the term “environment,” many assume that “environment” refers to a systemic perspective that focuses on the deep, existing connections among the sky, water reserves, and soil (ie, the global environment or the environment of specific geographic areas). In this viewpoint paper, the concept of environment refers to the consideration of all living and nonliving things in an ecosystem. This accounts for issues that are related to biodiversity conservation and the intimate links among the health, care, and well-being of all components in any given ecosystem [48].

The control and governance of environmental sustainability can be best approached by assessing ecosystem services that are capable of quantifying and valuing all the goods and services that are generated within the ecosystems themselves. Recently, such networks have been increasingly endowed with digital technologies such as (1) environmental management and monitoring information systems; (2) automated and scalable approaches for collecting, digitalizing, and assembling geocoded big data; and (3) information-fusion algorithms that use multiple data streams and clinical decision support algorithms that integrate population-based, public health–focused perspectives into outbreak detection–focused management systems [49-51].

Environment turns therefore out as “digital” in ecosystems that, under a digital vest, feature animals and humans, as well as software and robots, as autonomous and interacting agents to enhance decision support. The goals of digital ecosystem implementation include improving the efficiency of communication and avoiding peoples’ dependence on centralized or distributed control dynamics [52]. Such digital biodiversity originates from common substrates of data, information, and knowledge that are now accessible, available, and able to be analyzed in novel and innovative ways. However, this puts us into a Copernican situation [53], which is a prerequisite to figuring out appropriate digital solutions (ie, methods for safeguarding digital biodiversity) that are based on people’s intelligence and rationality. Such solutions are preferable over those that are gained through traditional artificial intelligence methods [52,54].

### The Three Perspectives as a Whole

In the broad digital ecosystem, ODH interventions must support, improve, and lead to efficient end-to-end processes for predictive, personalized, preventive, and participatory health care [42]. The management of disruptive innovation is the catalyst required for making this leap forward [55]. This requires the systematic, continuous, and intelligent integration of big, smart, and multidimensional data into digital health technologies. Such technologies are used for detecting, monitoring, and tracking the origins and causes of emerging pathogens, disease outbreaks, and disease-related trends that affect both humans and animals in the ecosystem. Thus, a challenging key issue that ODH must face is collecting and managing critical indicators that are related to human and veterinary health care, education, citizen engagement, environmental observation, and the health care industry. However, these indicators are required for active innovation. The COVID-19 outbreak is an example of an event that has triggered digital innovation and revolution in health care (ie, including ODH) on a global scale; the pandemic has quickly strengthened the overall care domain [56].

The three perspectives in the “individual-population-ecosystem” triad need to be further assessed as individual perspectives and as a specific set of strongly interconnected dimensions. These aspects will be discussed in-depth in the next sections.

## The Five Dimensions: The Essential Fabric of Interconnections

The dimensions layer provides an integrative view of ODH. This layer shows society’s outlook on the three perspectives and, in turn, the two keys. ODH depends on citizens' engagement and their ability to support and contribute to health care organizations in a continuously forward-moving environment.

### Education and Citizen Science

ODH should not be limited to higher education in health-related fields. Basic and transversal knowledge and an understanding of health and its digitalization must be provided by education programs for all levels and disciplines (ie, similar to how the COVID-19 pandemic needs to be understood at all levels). ODH is about more than supporting data management. ODH provides learning opportunities to citizens, so that they can protect and take care of themselves when they share personal data on social networks [57-59]. At its core, ODH teaches people how to differentiate between real and fake news, which are disseminated over the mass media and the internet.

The next generation of researchers and practitioners will be actively involved in redefining critical transition points for incorporating new knowledge into ODH, and constantly updating the underlying philosophy and the scientific, technological, and regulatory implications of ODH [60]. Fully appraising an ODH perspective is challenging due to its intrinsic complexity. Therefore, researchers, practitioners, and citizens need to engage in science and cocreate sound implications for health and well-being. The core challenges of ODH include (1) establishing a systemic and integrated understanding of the health and wellness of humans and animals in their common ecosystem; and (2) establishing how digital systems may support and improve the health and wellness of humans and animals. Therefore, it is necessary to provide learners with project-based learning and case-based learning, which are well-known methods that allow learners to autonomously develop their skills and form learning communities [61-64].

### Citizen Engagement

Supporting digital health literacy by actively encouraging everyone (ie, from children to elderly people) to engage with personal health, public health, and environmental monitoring systems can increase citizens’ awareness. Advancements in mobile and ubiquitous tracking, reporting, and follow-up apps/web-based systems have provided opportunities for citizens to engage and collaborate with governmental, health, environmental, and ecological organizations [65]. Therefore, opportunities in which citizens use their electronic health data to manage personal health and share personal information require citizens who trust in health care delivery. The use of implemented opportunities depends on patient engagement, the direct benefits for users, and health care management organizations [66]. Smart cities and neighborhoods (ie, cities and neighborhoods that extensively use smartphones that can geolocate individuals and trace/track behaviors) [67] can be intrusive sources of information. The act of intrusively obtaining information has introduced ethical dilemmas, challenged citizens’ trust, and disrupted societal norms. The European General Data Protection Regulation has been implemented to reduce the amount of excess data in the overall data sphere. Nevertheless, in cases of force majeure, intrusive approaches that are implemented during pandemics outbreaks (eg, the COVID-19 outbreak) may help public health decision makers understand how to develop efficient policies that are based on real-world and near–real-time data [68]. Therefore, policy decision makers may decide to passively involve the population by allowing the use and analysis of data on individuals, mass movements, and individuals’ (ie, humans’ and animals’) engagement with and access to certain facilities. This would mean having citizens engage with complex policy making over time (ie, for policies that are related to ethics, regulation, decisions on big smart data, and the shaping of social norms) [69]. From an ODH point of view, collaboration and transparency are essential for enhancing health systems’ and government authorities’ times to response during disruptive and potentially major events [45]. For example, in Taiwan, efficient smart contact tracing analyses were conducted to support an automated alert messaging system, which informed citizens about who required quarantine and isolation after coming into contact with the potentially infected passengers of the Diamond Princess cruise ship [70]. Furthermore, the European eHealth Network established the cross-border directive on patient’s rights to cross-border health services (ie, article 14), and recently developed guidelines for the interoperability of contact tracing apps [71]. These examples show how ODH views the health-related and environment-related movements of individuals. However, several countries have discontinued COVID-19 contact tracing apps after data protectorate organizations considered these apps to be too intrusive [72].

### Human and Veterinary Health Care

ODH aims to answer complex questions about zoonotic disease follow-ups by assessing public health impacts, surveilling human and animal health, and conducting related risk assessments and management processes [73,74]. Recently, health care delivery services have focused on providing services to clusters of patients [75]. The timely identification of health care customers, efficient service provision, and the continuous improvement of care quality accelerate the digital transformations of human and veterinary health care systems. In the context of the COVID-19 pandemic, environmental factors (eg, weather, pollution, and food) and animal health (eg, pets, livestock, and food) [76] influence human health, care, and well-being.

Digitalization and the increasing use of mobile and ubiquitous health/wellness apps and platforms have allowed for the assessment of patients (ie, human and animal patients) via dynamic, near–real-time methods that use patient-generated and participatory data. These methods enhance personalized health delivery services, such as preventive recommendations and predictive alert systems [77]. This new time- and space-oriented paradigm recalls connected health model features. It also allows us to view teleservices (ie, digital health services) as a full component of integrated care for health care customers and citizens who share their data to support the development of new health care practices and guidelines.

An example of connected health tools is SMS text messaging services, which are used to maintain patients’ involvement in their treatments (eg, vaccinations, rehabilitation, and medication renewals) by improving their adherence to therapy [75,78,79]. In terms of pets or livestock, owners can use connected health tools to stay involved in the health surveillance and follow-up of their animals [80]. In terms of the ODH framework, connected health tools support efforts for increasing global health security, as these tools help with collecting near–real-time feedback from populations and health care organizations [81].

### Digital Health Care Transformation 4.0

The health care system that deals with humans and animals is comprised of (1) health care service providers and insurers; (2) medical equipment and pharmaceutical product producers, sellers, deliverers, and maintenance service providers; (3) regulatory agencies and standardization organizations; and (4) health care customers, patients, and caregivers.

The entire health care sector is driven by digitalization and interconnectivity, which make up the foundation of new automation paradigms. The main challenges of the new health care framework include managing, collecting, storing, archiving, and analyzing a wide range of real-time data that are made available by any kind of organization and any kind of connected system. The leading goals of the current industrial revolution (ie, Industry 4.0) [82] are to enhance the automation and connectivity of systems, by increasing the interoperability and flexibility of systems and allowing for decentralized, real-time data collection and storage. The objective of Industry 4.0 is to use findable, accessible, interoperable, reusable, ethical, and revisable (FAIRER) data [83]. By adopting a FAIRER model, health care will benefit from greater traceability, flexibility, adaptability, and efficiency in health delivery processes [84]. As the health industry continues to adopt the ODH paradigm, a strong dependence on smart data means that standardization and interoperability framework issues are more critical than ever. A large number of systems need to be interconnected to collect real-time data that are generated by all parts of the health care system. The Health Level 7 Fast Healthcare Interoperability Resources standard [85] has been quite successful in this regard, as it focuses on the use of application programming interfaces within and across health/care sectors to improve a wide range of areas (ie, interconnectedness to contractual agreements).

The global health care industry has used the same technologies as those used by Industry 4.0 in the digital era [86], to achieve the use of the well-known “4.0” suffix. Telemonitoring systems use technologies that were initially designed for Industry 4.0. These technologies have been extensively used in human medicine [87,88]. Recently, their use has expanded as a result of the COVID-19 outbreak [89], as teleconsultation technology use has become the new norm in medical care. In terms of ODH, the same approach has been used by veterinarians. They can use Industry 4.0–related technologies to receive health and wellness information from wearable devices or implanted sensors that provide data on physiological signs. Veterinarians can also use these technologies to receive third-party, pet-related information [90]. The integration of these systems into pet health care may provide a better understanding of factors that affect pets’ health and pet owners’ benefits. Moreover, data on changes in a pet’s health can be used as pet owner health indicators [91] and environmental quality indicators [92].

3D printing is an emerging dynamic field in health care industries [93], including therapeutic industries (eg, printing prosthetics implants), human and veterinary care industries (eg, 3D printing in dental surgery), and educational industries [64]. The use of this on-demand technology supports the provision of personalized therapies and medical products to patients who are directly involved with health care practitioners. 3D printing has been shown to reduce the time to treatment by removing the need to travel back and forth between production laboratories and clinics.

### Environment

Environmental monitoring is a critical dimension of community-oriented management in day-to-day situations or disruptive situations, such as hazardous material incidents or disasters (eg, the COVID-19 pandemic) [94,95]. The digital landscape has allowed theoretical and laboratory-limited developments in automation and artificial intelligence (ie, those in the last several decades) to be put into practice [96]. Discussing the environment dimension of ODH means having to discuss the “human-nature-pollution” relationship and its role in the smart city paradigm. People who live in high-density urban areas have been increasingly using technological systems to report unusual events, thereby improving citizen engagement [97,98]. Furthermore, these people have been increasingly using green technologies, which reduce the negative impact of certain actions on the environment (eg, carbon dioxide emissions, car traffic, and waste production) and health [99].

With regard to the ODH, this environmental revolution means more than using smartphone and Internet-of-Things technologies for behavior, wellness, and health monitoring purposes. This revolution is a consequence of the Industry 4.0 revolution [100], which aims to enable citizens and their communities to interact with complex, but easy-to-use, digital systems. However, the Industry 4.0 revolution has different issues, which are mainly related to development costs, monitoring costs, implementation costs, maintenance costs, and the understanding and expectations of users (eg, citizens and decision-makers) [101].

The environment dimension is strongly related to the human and veterinary health care, One Health, and ODH. Wild and domestic animals are used as a part of surveillance platforms that provide early warnings about health hazards, which may impact the whole ecosystem. This is an example of why the environment, animals, and humans are part of the One Health “individual-population-ecosystem” triad [102]. Environment systems must be able to integrate, and be integrated into, health care management systems, to enrich data, information, and knowledge; actively support decision-making processes about environmental exposures and health risks; and disseminate efficient recommendations with the right communication tools [103]. This ODH dimension also relates to smart environment platforms that provide information to smart homes to monitor and deliver personalized services to older adults [104].

## Digital Technologies as a Catalyst for the Integration of Keys, Perspectives, and Dimensions

### Technology as a Catalyst for “ODHness”

The main aims of digital health are improving health,  care, wellness, and public health through BMHI research [22]; digital health expands these concepts by allowing for the consideration of digital consumers who use a wide range of smart-devices, connected medical equipment, or connected wellness equipment. Digital health also encompasses other digital technologies for health, such as the Internet of Things, artificial intelligence, big data, and robotics. These technologies have become a full component of day-to-day life, [84] and in some cases, health-related crisis management [46,70]. Healthy individuals, chronic patients, and health management organizations have been increasingly using automated reminder systems, smartphone apps, and health monitoring wearables [75,105,106]. In terms of ODH, technology serves as the catalyst for digitizing One Health information for ODH; advanced technological innovations have emerged to improve citizen engagement and empower future health ecosystems (Figure 1).

In health crises like the COVID-19 pandemic, the applications of digital technologies (eg, managing, preparing for, and mitigating crises) include the planning and scheduling of response and recovery processes. Digital technologies use surveillance, contact tracing, contact tracking, testing, confinement, and other health and care methods when necessary (eg, after ethical and legal implications have been considered) [107]. A similar level of attention is necessary for effectively tracking veterinary information that needs to be extracted from vast amounts of data. This process involves the integration of animal medical data into real-time information systems that are dedicated to supporting public health [108]. Thus, the role of veterinarians and veterinary informatics enhances that of BMHI (ie, protecting health care), as veterinarians have been called on to help deal with the increase amounts of dynamic data on emerging and reemerging infectious diseases. Local and supranational indicators of health have been used to identify global-level risk factors and causes of health problems that arise at the human, animal, and environmental levels, which need to be taken into account [109,110].

According to the WHO vision, establishing an interoperable digital health ecosystem that is capable of seamless, secure health data exchange and processing is crucial for combating pandemic outbreaks. Developing infrastructures and apps that allow the use of health data to manage adverse events is within the scope of achieving the 17 health-related sustainable development goals that were issued in the WHO global action plan [111]. We therefore introduced the concept of digital One Health interventions [112] in this viewpoint paper as a set of digital functionalities that should be designed and deployed to (1) support specific initiatives that address human, animal, and environmental systems’ needs and challenges; and (2) assess, study, and collect data on these systems’ expected outcomes, unexpected outcomes, and effects [36]. This related to the selection of timely metrics for the outcomes of multicriteria decision analyses.

These digital functionalities can be also called “digitalities,” as they account for how technology has been embedded into human experiences and humans’ daily lives. This has resulted in core issues in social and cultural anthropology (ie, digital humanities) [112]. Digitalities also account for how humans affect animals’ daily lives, animal health issues, and human-animal relations. These digitalities aim to increase animal welfare (ie, digital animalities) [113]. Furthermore, digitalities aim to manage the complex web of human, animal, and environmental interconnections, by improving environmental governance (ie, digital environmentalities) [114,115].

The delivery of digital One Health interventions and the mechanisms of the impacts and contextual factors of these interventions can be referred to as “ODHness.” This term is based on the combined digitalities of the three overarching, complementary perspectives. These digitalities apply to the five ODH dimensions and their constituent subcategories. Therefore, the technology ring in Figure 1 serves as the connection between the ODHness concept and relevant digitalities.

**Figure 2:**
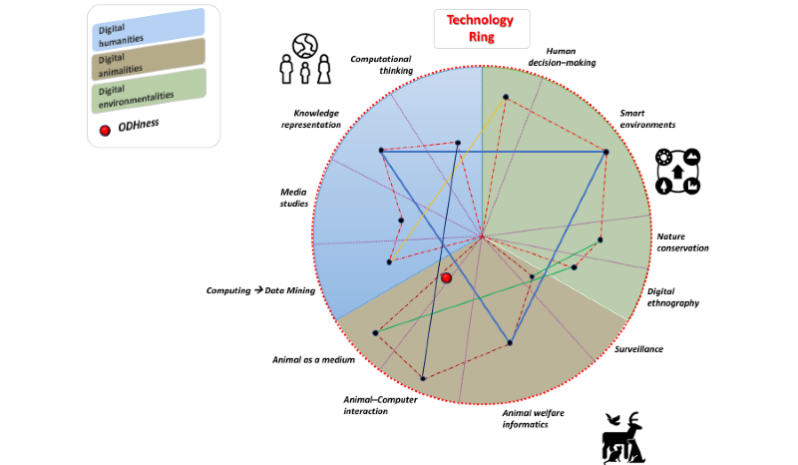
An example of the interactions within One Digital Health, which are based on the use of technology as a catalyst.

Figure 2 shows several topics that are relevant to each of the three digitalities (ie, the three colored areas inside the technology ring). Each topic is characterized by each portion within the corresponding colored area; the width of these areas depends on the number and the nature of the area’s related subtopics. It should be noted that these subtopics have not been clearly represented in the figure to ensure that people comprehend the ODH scheme. In terms of digitality, a specific metric or set of metrics must be used to evaluate the levels of development, use, contribution for each digital One Health intervention topic. These levels are represented with black dots for each subdomain, connected with each other via dashed lines. Since interactions between topics from different digitalities are possible, the edges that connect the different areas are represented with different colors. In addition, “trialities” (ie, triangles that connect all three digitalities) are represented via thick lines. Furthermore, each edge is assigned an appropriate weight. This of course means that harmonization must exist between the implemented metrics. Harmonization is required to fulfill the needs of a multicriteria decision analysis.

**Figure 3:**
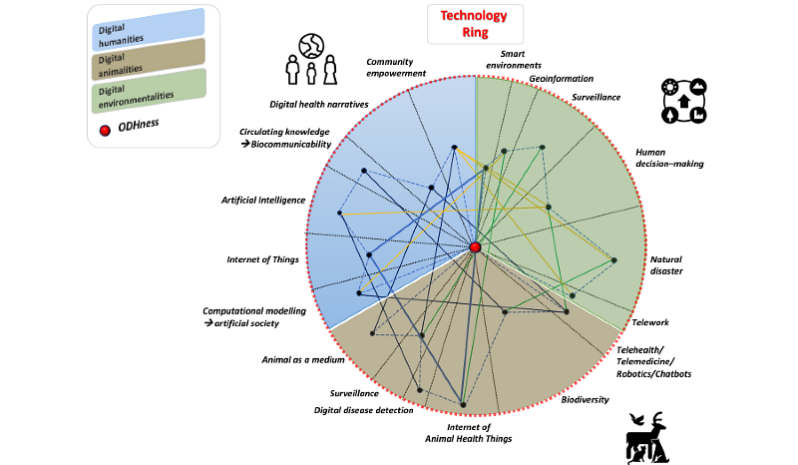
Pandemic-related "ODHness" interactions within the technology ring.

The technology ring in Figure 2 encompasses the set of digital functionalities that the ODH framework relies on. Therefore, technology acts as the catalyst that connects and unifies the ODH disciplines. ODHness is represented by the technology ring’s “center of mass” (ie, the red dot in Figures 2 and 3). More specifically, the “center of mass” is the unique point where the weighted relative position of the distributed mass (ie, the edges) equals 0. In other words, this means that a simple set of digital technologies (ie, those related to the human, animal, and environmental fields) is not capable of providing timely solutions in an interdisciplinary context, unless these technologies act as the catalyst for balancing the different perspectives, needs, and interests of ODH. In Figure 2, we attempted to highlight the “human-animal-environment” computer interaction research area, which includes technology designs that support animals in different contexts, and the development of user-centered approaches for designing technologies that are intended for more-than-human animals [116].

Examples of possible pandemic-related interactions within the technology ring are shown in Figure 3. Several of the main topics of each digitality are shown. The levels of development, use, and contribution for each digital One Health intervention are also shown. For convenience, the ODHness point is represented by the center of the technology ring.

## Discussion and Conclusions

ODH is a framework that integrates key dimensions from two established fields—digital health and One Health. The latter focuses on the interconnectedness of human, animal, and environmental health research. The former focuses on providing tools and domain expertise to support health practitioners in their administrative (eg, consultation scheduling), clinical (eg, necessary tests, drug-drug interaction alerts, and specific follow-up reminders) and scientific (eg, disease prevalence analysis, data collection, and reporting) activities. The digital health field also encompasses citizens, who are the consumers of health services (eg, healthy individuals, sick individuals, relatives who assist sick individuals in day-to-day life, and insurers).

ODH analyzes the health data and information of digital health ecosystem components, because the intents, processes, and products of these components (ie, health, care, and wellness) are integrated into health ecosystems. Moreover, ODH analyzes three perspectives. These perspectives include three different areas that deal with the growing scientific evidence and perspectives that come with technologies that are meant to support health, care, and well-being activities in separate ecosystems. Furthermore, this unified framework for future health ecosystems analyzes the impact of five dimensions (eg, education provides short-, mid-, and long-term learning opportunities for improving citizens’ engagement with human and veterinary health care services; environment monitoring; and health care industry–developed, interoperable early warning systems).

The COVID-19 pandemic has increased the world’s understanding of the close relationships among the environment, animals, and humans [117]. Therefore, it is more critical than ever to effectively manage the pandemic by assessing the innovative two keys, three perspectives, and five dimensions in the unifying ODH framework. It is also important to assess the pandemic’s impact on health ecosystems by facilitating systematic collaboration among animal and environmental scientists, health care practitioners, citizens, governments, academics, and industrial manufacturers.

The next step in developing and testing the ODH framework will be performed by the proposed EFMI ODH working group. The proposed EFMI ODH working group will elaborate upon the ODH framework, validate the methodology, and evaluate the ODH. They will also examine specific ODH scenarios in-depth and analyze the relationships between the perspectives in the ODH framework and BMHI dimensions (ie, data, information, and knowledge). Analyzing these relationships is expected to yield new insights and support an integrative, systemic, and syndemic decision-making process that can be assessed from a multidisciplinary perspective [117,118]. We expect that this process will be instrumental in shaping future health ecosystems, providing novel learning opportunities for citizens, and educating the next generations of BMHI practitioners.

## Abbreviations

BMHI: biomedical and health informatics
EFMI: European Federation for Medical Informatics
FAIRER: findable, accessible, interoperable, reusable, ethical, and reversible
ODH: One Digital Health
WHO: World Health Organization
